# Reduction of GAS5 and FOXD3-AS1 long non-coding RNAs in patients with bipolar disorder

**DOI:** 10.1038/s41598-023-41135-z

**Published:** 2023-08-24

**Authors:** Bita Zamani, Mahdieh Mehrab Mohseni, Bahar Naghavi Gargari, Mohammad Taheri, Arezou Sayad, Zeinab Shirvani-Farsani

**Affiliations:** 1 Department of Cell and Molecular Biology, Faculty of Life Sciences and Technology, Shahid Beheshti University, Tehran, Iran; 2 Department of Genetics, School of Medicine, Shahid Beheshti University of Medical Sciences, Tehran, Iran; 3 Department of Basic Sciences, School of Nursing and Midwifery, Shahid Beheshti University of Medical Sciences, Tehran, Iran; 4 Institute of Human Genetics, Jena University Hospital, Jena, Germany; 5 Department of Medical Genetics, School of Medicine, Shahid Beheshti University of Medical Sciences, Tehran, Iran

**Keywords:** Genetics, Molecular biology, Biomarkers

## Abstract

Bipolar disorder (BD) patients suffer from severe disability and premature death because of failure in prognosis, diagnosis, and treatment. Although neural mechanisms of bipolar have not been fully discovered, studies have shown long noncoding RNAs (lncRNAs) can play an important role in signaling pathways such as PI3K/AKT pathway. There has been little study on deregulated lncRNAs and the lncRNAs’ mode of action in the BD. Hence, we aimed to investigate the expression of PI3K/AKT pathway-related lncRNAs named TUG1, GAS5, and FOXD3-AS1 lncRNAs in the PMBC in 50 bipolar patients and 50 healthy controls. Our results showed that FOXD3-AS1 and GAS5 under-expressed significantly in bipolar patients compared to healthy controls (P = 0.0028 and P < 0.0001 respectively). Moreover, after adjustment, all P values remained significant (q value < 0.0001). According to the ROC curve, AUC (area under the curve), specificity, and sensitivity of these lncRNAs, GAS5 and FOXD3-AS1 might work as BD candidate diagnostic biomarkers. Taken together, the current results highlight that the dysregulation of FOXD3-AS1 and GAS5 may be associated with an increased risk of BD.

## Introduction

Bipolar disorder (BD) patients all over the world suffer from severe disability, premature death, and suicide because of failure in on-time prognosis, diagnosis, and treatment^1^. BD patients experience a kind of severe mood swings between depression and happiness^2^. The biological mechanisms underlying BD remain largely unclear, although some researchers claim that dysfunction of several signaling pathways including PI3K/AKT (Phosphatidylinositol 3-kinase/AKT) can affect it. Reduction of Akt-mTOR signaling has been demonstrated in a subset of BD subjects^3^. This PI3K/AKT signaling pathway is required for cell survival of multiple neurons^4^. One of the functional factors with many important roles in adjusting the PI3K/AKT signaling pathway is long noncoding RNAs (lncRNAs)^5^. In addition, several lncRNAs have been reported to be involved in the pathogenesis of BD^6^. However, according to our literature review, there has been little study on deregulated lncRNAs in BD^6–8^ and the lncRNAs’ mode of action in the BD etiology is still largely obscure. In the present study, based on the previous findings of PI3K/AKT signaling pathway dysregulation in BD patients^3,4^**,** we analyzed the expression of three PI3K/AKT-related lncRNAs (TUG1, GAS5, and FOXD3-AS1) in BD patients compared with healthy controls. lncRNA taurine upregulated gene 1 (lncRNA TUG1) reduced TGF-β1 expression and suppressed the activation of the PI3K/AKT pathway in mesangial cells^9^. Qi et al. found that TUG1 elevated pulmonary fibrosis progression by inducing inflammation and activating PI3K/AKT/mTOR pathway^10^. In addition, TUG1 is highly expressed in the adult brain and participates in multiple processes associated with nervous system diseases^11,12^. For example, TUG1 under-expression significantly improves balance in patients with Parkinson's disease and prevents the expression of infectious factors^13,14^. The other lncRNA is growth arrest-specific 5 (GAS5) which was deregulated in neuronal diseases like epilepsy and its silencing prohibited the KCNQ3 expression via miR-135a-5p sponging to block the epilepsy progression^15^. Meanwhile, Liu et al. indicated that GAS5 activates the PI3K/AKT/mTOR signaling pathway, resulting in inhibited cell proliferation and metastasis in laryngeal cancer^16^. Forkhead box D3 antisense 1 (FOXD3-AS1) is another lncRNA that regulates cerebral ischemia/reperfusion injury^17^. Moreover, FOXD3-AS1 as an oncogenic lncRNA induces the occurrence and development of glioma by regulating FOXD3 expression^18^. FOXD3-AS1 was also shown to induce PI3K/Akt pathway and increase the expression of TFF1, leading to cell proliferation^19^. Hence, we anticipated that the investigation of lncRNA expression in BD improves our understanding of its neurological pathways and pathogenesis. 

## Materials and methods

### Subjects

The current investigation was performed using blood samples from 50 BD outpatients and 50 age, sex, and ethnically matched healthy controls. All patients were evaluated according to the presence of manic and depressive episodes based on the Diagnostic and Statistical Manual of Mental Disorders-5 by the official psychiatrist of Emam Hosein Hospital. None of the BD patients or healthy controls had a history of neurological, metabolic, or autoimmune disorders. The possible effects of other confounding factors such as treatment, symptom severity, smoking, and a previous number of ECT sessions were adjusted, too. Written informed consent forms were signed by all participants. The investigation protocol was approved by the ethical committee of the Shahid Beheshti University of Medical Science (IR.SBMU.MSP.REC.1400.620).

### RNA extraction and cDNA synthesis

Peripheral blood was collected from mentioned patients and controls, then peripheral blood mononuclear cells (PBMC) were isolated by Ficoll-Paque™ for total RNA extraction using the RNAX kit. The extracted RNA was treated with DNase I for 30 min at 37 °C. RNA quantity and quality were evaluated by UV spectrophotometry and agarose gel. cDNA was synthesized by RT-PCR Pre-Mix Kit according to the kit instructions (3 μg of pure total RNA, oligo (dT) and random hexamer primers, and the Revert Aid™ Reverse Transcriptase was applied in a final volume of 20 μl PCR mixture).

### Quantitative real-time PCR

Quantitative real-time PCR was performed on the ABI 7500 sequence detection system (Applied Biosystem, Foster City, CA, USA) using 7.5 μl of BIOFACT™ 2X Real-Time PCR Master Mix, 0.5 μl cDNA and 0.8 μl of each primer. The PCR was performed as follows: an initial denaturation for 5 min at 95 °C followed by 40 cycles of denaturation for 10 s at 95 °C, annealing for 30 s at 60 °C, and extension for 20 s at 72 °C. ΔCt (ΔCt = Ct _target gene_ – Ct _reference gene_) for cases and controls were calculated and the fold change of genes’ expression was measured by 2^−ΔΔCt^ (ΔΔCt = ΔCt _cases_ – ΔCt _controls_). GAPDH was used as an internal control gene for comparisons. Table 1 displays the information on primers and amplified sections.

**Table 1:** Primers used in RT-qPCR.

Gene name	Sequence (5′→3′)	Primer size	Product length
FOXD3-AS1	F: CCTCCAAGATTTAACTTCCAA	21	153
	R: ACAGACAGGGATTGGGTTC	19	
GAS5	F: CACAACAAGCAAGCATGCAG	20	169
	R: TCTTCTTGTGCCATGAGACTCC	22	
TUG1	F: GCTCTCTTTACTGAGGGTGCTT	22	304
	R: GGATCTGTCAAGTCTCAATGTTGG	24	
GAPDH	F: CCATGAGAAGTATGACAAC	19	115
	R: GAGTCCTTCC ACGATACC	18	

### Statistical method

GraphPad Prism 9 (GraphPad Software, Inc., San Diego, CA, USA) software was used for statistical analysis. The data normality was examined using the Kolmogorov–Smirnov test. The student’s t-test was used to compare the difference in expression levels of each lncRNA between patients and controls. Pearson's correlation test was used to assess the association between various lncRNA expressions. The receiver operating characteristic (ROC) curves were performed to investigate the specificity and sensitivity of aimed genes and lncRNAs as potential biomarkers for BD diagnosis. P < 0.05 was considered a significant difference.

### Ethics approval and consent to participant

All procedures were in accordance with the ethical standards of the national research committee and with the 1964 Helsinki Declaration. Informed consent forms were obtained from all study participants. The study protocol was approved by the ethical committee of Shahid Beheshti University of Medical Sciences.

## Results

The fifty BD patients and 50 healthy controls who participated in this study are summarized in Table 2.

**Table 2:** Demographic and clinical features of BD patients and controls.

Clinical characteristics	NO	Female: male	Mean age (year)	Age range (year)	Disease duration (year)	Age of onset
Cases	50	23:27	40.72 ± 8.2	20–56	11.69 ± 3.4	28.1 ± 6.2
Controls	50	30:20	34.08 ± 5.8	17–54	–	–

### Gene expression analyses

**Figure 1:**
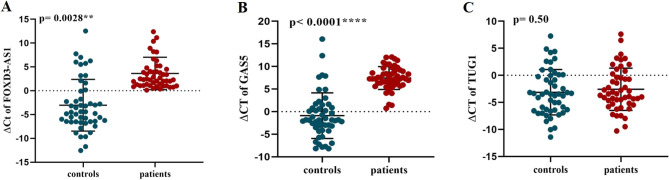
The ∆Cts of lncRNAs FOXD3-AS1 (**A**), GAS5 (**B**), and TUG1 (**C**) in the blood samples of BD patients and control subjects. **P < 0.01, ****P < 0.0001.

Our study showed that the expression levels of FOXD3-AS1 (P = 0.0028) and GAS5 (P < 0.0001) were significantly different between BD patients and healthy individuals (Fig. 1A and B). In BD patients, the FOXD3-AS1 expression reduced 5.3 times and the GAS5 expression decreased 33.3 times compared to healthy individuals. In addition, after adjustment, all P values remained significant (q value < 0.0001 for both lncRNAs). However, the difference in expressions of lncRNA TUG1 was not statistically significant between the two groups (P = 0.50) (Fig. 1C).

**Figure 2:**
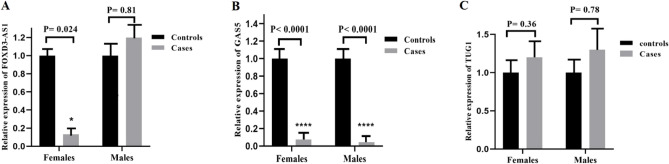
The expression analysis of lncRNAs FOXD3-AS1 (**A**), GAS5 (**B**), and TUG1 (**C**) in patients and controls based on gender. *P < 0.05, ****P < 0.0001.

Furthermore, the lncRNA FOXD3-AS1 (P = 0.024) was significantly under-expressed in female BD patients (7.5 times) compared to female controls. However, the expressions of lncRNA FOXD3-AS1 (P = 0.81) were statistically non-significant between the two male groups (Fig. 2A). A significant difference in the GAS5 expression was seen between female patients and female controls (P < 0.0001) as it reduced 21 times in BD females compared to control females (Fig. 2B). In addition, GAS5 was significantly down-regulated (65 times) in male BD patients compared to male controls (P < 0.0001) (Fig. 2B). Moreover, the expressions of TUG1 were not statistically significant among two female groups (P = 0.36) or male groups (P = 0.78) (Fig. 2C).

### ROC curve analysis

**Figure 3:**
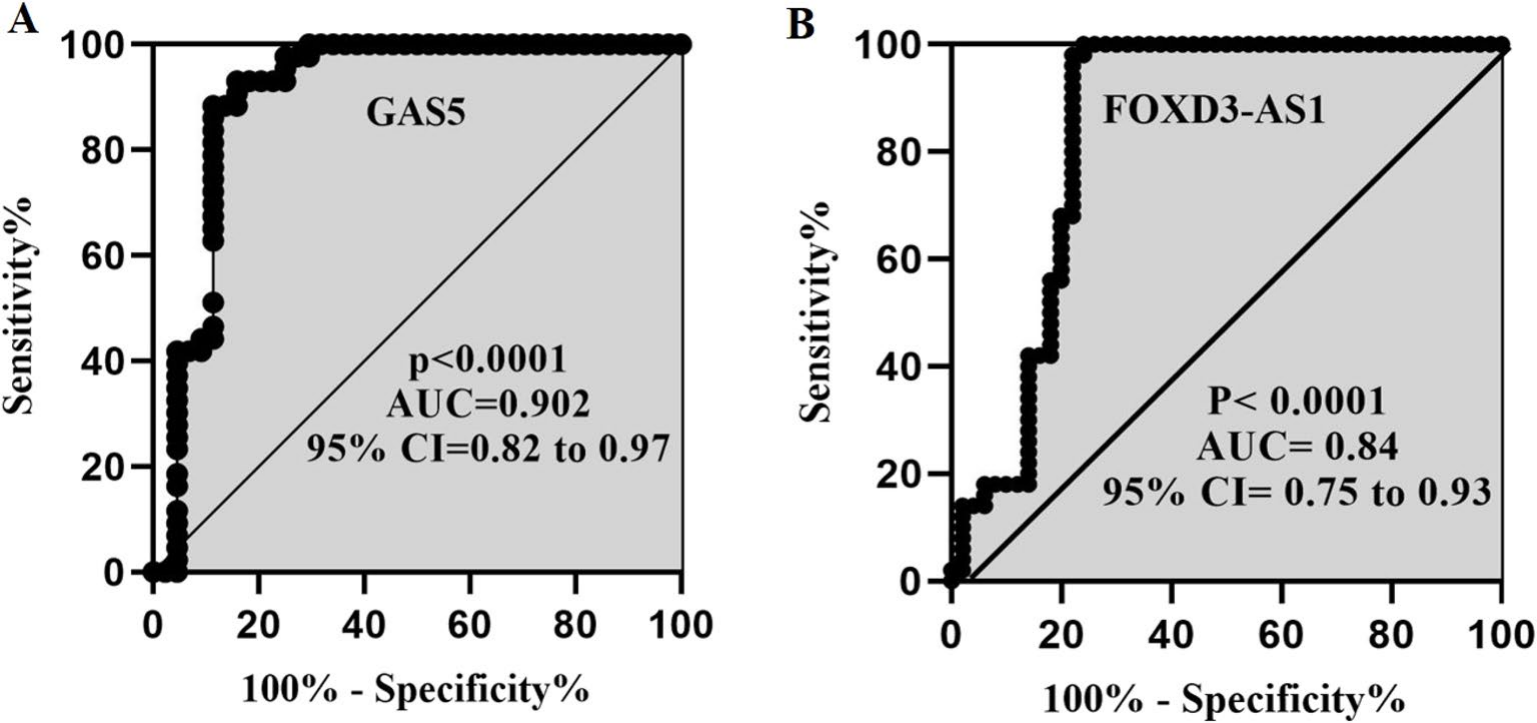
ROC curve analysis of GAS5 (**A**) and FOXD3-AS1 (**B**). AUC: area under the curve.

Using ROC Curve analysis, the sensitivity and specificity of the expression levels of the studied lncRNAs were evaluated. The results showed that GAS5 with an area under the curve (AUC) 0.90 and P-value < 0.0001 (sensitivity = 94%, specificity = 86%, and cut-off = 3.53), as well as FOXD3-AS1 with AUC 0.84 and P-value < 0.0001 (sensitivity = 88%, specificity = 78%, and cut-off = 0.84), could differentiate well between patients and controls (Fig. 3A and B). Therefore, according to AUC, GAS5 lncRNA had better performance in differentiating disease status in BD patients compared with another lncRNA.

### Correlation analysis

The results of pairwise correlation analysis between expression levels of three lncRNAs showed that there is no significant correlation between the expressions of these lncRNAs with each other (Table 3). There is also no significant relationship between the age, disease duration, and age of onset of patients and expression levels of TUG1, GAS5, and FOXD3-AS1 lncRNAs (Table 3).

**Table 3:** Pairwise correlation between expression levels of lncRNAs in cases group, correlation between expression levels of lncRNAs and demographic data of patients.

Variables	FOXD3-AS1		GAS 5		TUG 1	
	r	P-value	r	P-value	r	P-value
Age	− 0.02	0.88	0.08	0.60	− 0.27	0.08
Disease duration	− 0.002	0.98	0.019	0.90	0.17	0.24
Age of onset	− 0.23	0.11	− 0.17	0.27	0.13	0.37
TUG 1	0.21	0.18	− 0.18	0.24	–	–
GAS 5	0.18	0.24	–	–	–	–

## Discussions

Approximately 80% of BD people commit suicide or suffer from suicidal thoughts^20^ as a result of a lack of appropriate diagnosis or treatment. Due to BD’s comorbidity with other mental illnesses as well as different symptoms in different patients, its diagnosis seems very difficult^2,21^. Although biomolecular neural mechanisms of BD have not been discovered, recent studies have shown genetic factors such as long non-coding RNAs can play an important regulatory role in neurological pathways such as PI3K/AKT in other neural diseases^22,23^.

In this study, for the first time, we evaluated the expression levels of TUG1, GAS5, and FOXD3-AS1 lncRNAs in BD patients and healthy controls, which all are involved in PI3K/AKT signaling pathways and neurological processes.

Investigations on neural diseases bold the role of GAS5 lncRNA in signal transduction regulation. For instance, GAS5 activation induces the PI3K/AKT signaling pathway, which protects hippocampal neurons from damage by regulating miR26-a/EGR1. In mice with depression-like behaviors, GAS5 binds specifically to miR26-a to keep EGR1 active, consequently, EGR1 deactivates the PI3K/AKT pathway, subsequently, infectious factors are released and apoptosis of hippocampal cells occurs. As a result, GAS5 silencing prevents apoptosis of hippocampal neurons^24^.

Another neural disorder is Cerebral Ischemia (stroke) which causes severe damage to neurons. Examination of the brain cells of ischemia people showed the GAS5 and PTEN RNA overexpression but the miR-21 underexpression. GAS5 inhibits the miR-21 expression and thus increases PTEN which inactivates the PI3K/AKT signaling pathway, so shutting down GAS5 maintains neuronal health through the PI3k/AKT signaling pathway and seems an appropriate treatment for cerebral ischemia^25^.

Glioma as another neural condition shows severe GAS5 downregulation compared to normal glial cells. GAS5 lncRNA targets GSTM3, so it is expressed in glioma cells compared to glial cells. GAS5 overexpression inhibits cell proliferation and migration while promoting cell death^26^.

**Figure 4:**
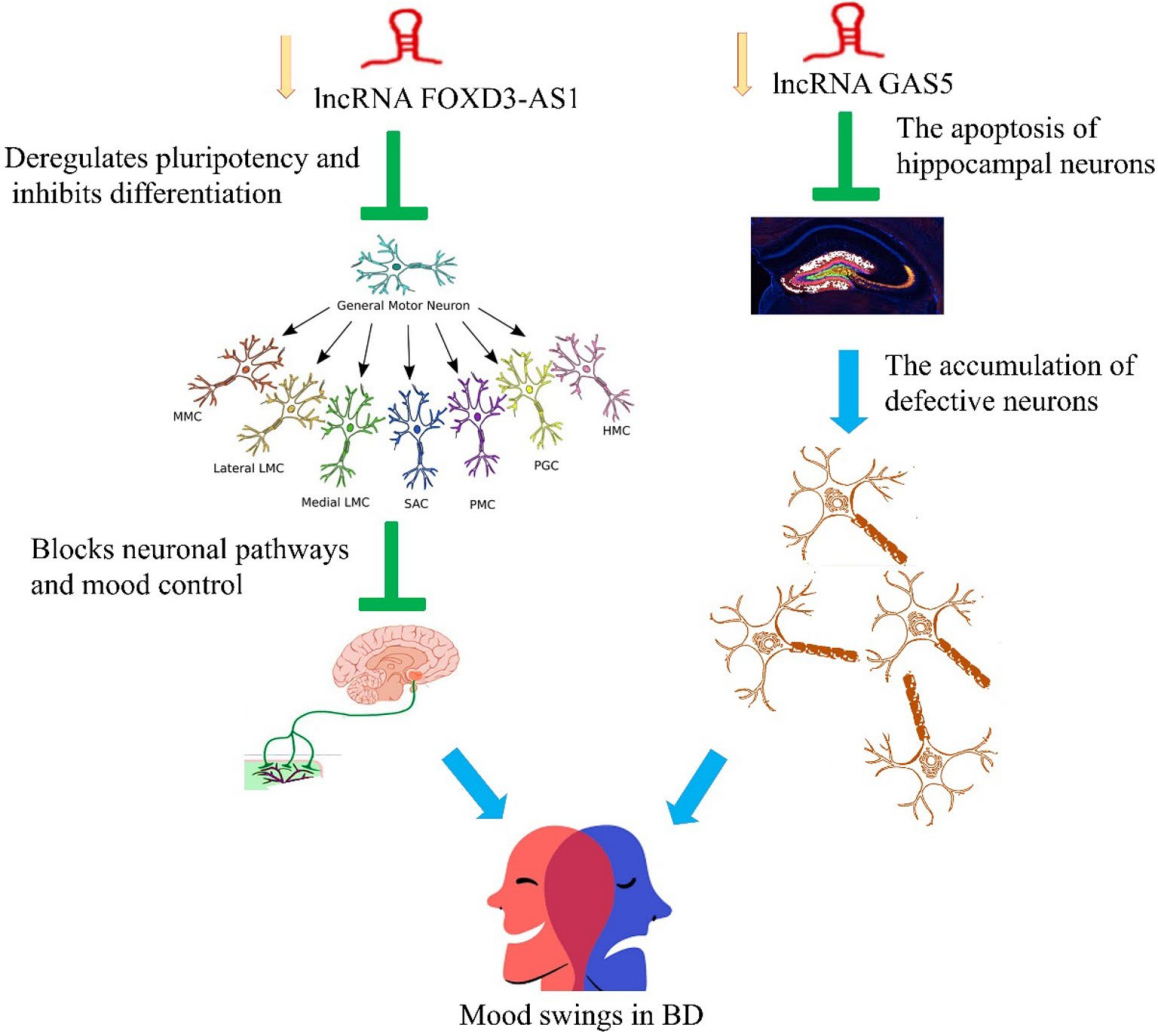
The potential mode of action of FOXD3-AS1 and GAS5 lncRNAs.

Based on our data the expression of GAS5 lncRNA in BD patients has decreased 33.3 times compared to healthy individuals. Therefore, GAS5 downregulation may inhibit the apoptosis of hippocampal neurons. According to other studies, these neurons have defects in bipolar individuals^27,28^ that must be eliminated through apoptosis, but in bipolar individuals, apoptosis of neurons may decrease because of lower levels of GAS5, resulting in the accumulation of these defective neurons in the bipolar brain and therefore causes mood swings (Fig. 4).

However, this hypothesis must be proven by further studies. The results obtained from the ROC biomarker assay showed 0.9 for the AUC of the GAS5 lncRNA. As this gene presents a sensitivity of 94% and a specificity of 86%, it is considered the potent BD diagnostic biomarker.

FOXD3-AS1 belongs to a category of promoter upstream transcripts (PROMPTs) lncRNAs with expression and functions related to the adjacent protein-coding transcripts. According to mechanistic studies, FOXD3-AS1 promotes cell proliferation, apoptosis, invasion, migration, chemotherapeutic resistance, and cell stemness by regulating the PI3K/AKT signaling pathway^29^. For instance, FOXD3-As1 has been found to inhibit epithelial-mesenchymal transition (EMT) and invasion via the induction of PI3K/Akt pathway and the miR-150/SRCIN1 axis in NSCLC A549 and H1229 cells^30^. So, FOXD3-AS1 silencing inhibits not only cell proliferation and migration, but interferes with the cancer cell line^31^. FOXD3-AS1 also plays an important role in nervous system tumors such as neuroblastoma and glioma. Based on cell cycle analysis, FOXD3-AS1 silencing in glioma cells prevented S/G2 cell cycle transition which inhibited cell proliferation^18,32^. Therefore, FOXD3-AS1 over-expression promotes neuronal differentiation and prohibits the growth, invasion, and metastasis of the neuroblastoma cells^32^.

FOXD3-AS1 can also induce pluripotency of human embryonic stem cells (hESCs). Undifferentiated hESCs over-expresses FOXD3-AS1, while endoderm and mesoderm differentiation is correlated with low FOXD3-AS1 expression. Down-regulation of FOXD3-AS1 results in pluripotency deregulations through the inhibition of endoderm pathways^33^.

Another research identified that FOXD3-AS1 increased neuronal apoptosis through miR-765/BCL2L13 and may be a potential biomarker for ischemic stroke^17^. Indeed, FOXD3-AS1 knockdown inhibits neuronal apoptosis and cerebral infarction and facilitates the recovery of neuronal function, thus exerting neuroprotective effects in ischemic stroke^29^.

Furthermore, FOXD3-AS1 is downregulated in neuroblastoma and allergic rhinitis presenting a protective role. In neuroblastoma IMR32 and BE (2)-C cells, FOXD3-AS1 inhibits PARP1 and CTCF, which leads to growth, differentiation, and invasion blockage^32^.

Our results showed that FOXD3-AS1 is significantly reduced (5.3 times) in BD patients compared to healthy individuals. The reduction of this lncRNA can deregulate pluripotency and inhibit differentiation therefore, many neuronal cells and tissues in the peripheral nervous system with essential roles in neuronal pathways and mood control will not be established correctly, this can cause irrational mood swings and other neurological symptoms attributed to BD patients (Fig. 4). Our ROC biomarker test showed an AUC of 0.84, a sensitivity of 88%, and a specificity of 78% for the FOXD3-AS1. Therefore, we can introduce this lncRNA as a BD-potent diagnostic biomarker.

Some studies have shown various TUG1 functions. For instance, TUG1 lncRNA works as an inhibitor of human glioma tumors^34^. TUG1 also acts as a ceRNA (Competing endogenous RNA) for miR-145 by directly binding to miR-145, reversing the function of TUG1 siRNA on AQP4 (Aquaporin4) effectively. AQP4 reduces OGD/R1 loss which decreases cell damage, so, it could provide a new therapeutic target in cerebral ischemia injury^11^. Some studies have shown that TUG1 is highly expressed in Parkinson's disease and its decrease prevents cell proliferation and the release of IL-1B, IL-6, and TNF-α factors^13^. In addition, TUG1 downregulation reduces oxidative stress, apoptosis, neuroinflammation, and pathological damage in SH-SY5Y cells of Parkinson's model by the miR-152-3p and PTEN under-expressions^13^. TUG1 was also shown to promote osteosarcoma proliferation, migration, and invasion via miR-219a-5p/PI3K/AKT signaling pathway. Moreover, the induction of the PI3K/AKT pathway enhanced the expression of TUG1 by promoting FOXM1 expression, resulting in a positive feedback loop in osteosarcoma^35^. In our study, the TUG1 gene was not significantly different in patients compared to healthy individuals.

In brief, the result of this study provided clues for an association between dysregulation of FOXD3-AS1 and GAS5 lncRNAs and bipolar disorder. However, this study faced limitations such as a small sample size. So, we highly recommend proving these valuable outcomes with more functional studies for clarifying the mechanism of their contribution to the etiology of BD pathology (Supplementary Table 1).

### Supplementary Information

Supplementary Table 1.

## Data Availability

The datasets used and/or analyzed during the current study are available from the corresponding author upon reasonable request.
