# Supplementary material for: Reduction of GAS5 and FOXD3-AS1 long non-coding RNAs in patients with bipolar disorder
Source: Sci Rep. 2023 Aug 24;13:13870. doi: 10.1038/s41598-023-41135-z (PMC10449891; doi:10.1038/s41598-023-41135-z)
Supplement: Supplementary file 1 — Supplementary Table 1. [file 41598_2023_41135_MOESM1_ESM.docx]

**Supplementary Table 1** Demographic data of study participant (Cases and Controls).

|  | **BD patients** | **Control** |
| --- | --- | --- |
| **Number** | 50 | 50 |
| **Sex**  **Female%**  **Male%** | 15 (30%)  35 (70%) | 15 (30%)  35 (70%) |
| **Mean age ±SD (range)** | 36.5± 10.2 (17-56) | 34.6± 9.5 (14-52) |
| **Disease duration ±SD (range)** | 3.86± 1.2 (1-9) | - |
| **Age at onset ±SD (range)** | 32.64± 8.5 (15-48) | - |
